# Marginal gap and internal fit of 3D printed versus milled monolithic zirconia crowns

**DOI:** 10.1186/s12903-023-03184-8

**Published:** 2023-07-04

**Authors:** Ashraf Refaie, Ahmed Fouda, Christoph Bourauel, Lamia Singer

**Affiliations:** 1 Oral Medicine Technology, University Hospital Bonn, Bonn, Germany; 2 Department of Fixed Prosthodontics, Faculty of Dentistry, Fayoum University, Faiyum, Egypt; 3 Faculty of Oral and Dental Medicine, British University in Egypt, Cairo, Egypt; 4 Department of Orthodontics, University Hospital Bonn, Bonn, Germany

**Keywords:** Yttria-stabilized zirconia crowns, 3D printing, Subtractive manufacturing, Marginal gap, Internal fit

## Abstract

**Background:**

This study aimed to evaluate and compare the marginal gap using two different methods and the internal fit of 3D printed and zirconia crowns.

**Methods:**

3Y-TZP zirconia crowns (*n* = 20) were manufactured using subtractive milling (group M) and 3D printed (group P). The marginal gap was measured at 60 points using vertical marginal gap technique (VMGT). On the other hand, the silicone replica technique (SRT) was used to evaluate the internal fit and was divided into 4 groups: marginal gap, cervical gap, axial gap, and occlusal gap where the thickness of light impression was measured at 16 references. The numerical data was tested for normality using Shapiro–Wilk's test. They were found to be normally distributed and were analyzed using an independent t-test.

**Results:**

Using VMGT, group P had significantly higher mean marginal gap values of 80 ± 30 µm compared to group M = 60 ± 20 µm (*p* < 0.001). Also, with the SRT, the marginal gap of group P (100 ± 10 µm) had significantly higher values compared to group M (60 ± 10 µm). The internal fit showed significant difference between the tested groups except for Axial Gap.

**Conclusions:**

Although milled crowns showed better results. The 3D printed zirconia crowns offer clinically acceptable results in terms of marginal adaptation and internal fit. Both VMGT and SRT are reliable methods for the assessment of the marginal gap.

## Background

Monolithic zirconia restorations are among the most commonly used treatment options in modern dentistry owing to their biocompatibility, strength, and esthetics [1]. The conventional technique for the fabricating zirconia crowns is subtractive milling through computer-aided design and computer-assisted manufacturing (CAD/CAM). Although subtractive milling technique is a very reliable method, it has many drawbacks such as the high production costs, the wear of the milling burs, the amount of wasted material, and the difficulty of milling complex geometries [2]. To overcome these drawbacks, many attempts have been made to manufacture dental crowns using additive manufacturing technologies (AM), so-called 3D printing techniques. AM techniques include selective laser sintering (SLS), selective laser melting (SLM), stereo-lithography (SLA), ink-jet printing (IJP), fused deposition modeling (FDM), and others [3].

Lithography-based Ceramics Manufacturing technique (LCM) could be used for the fabrication of ceramics. The CAD file of the desired design is virtually divided into very thin layers, the slurries (photosensitive ceramic suspensions) are deposited and cured using digital light processing (DLP), where the projected light source is used to cure the entire layer at once and the polymer network serves as a binder between the ceramic. The produced green bodies are a composite of polymerized binder with dispersed ceramic particles within, which is then cleaned with a direct stream of compressed air and suitable cleaning solvents to remove any excess or uncured raw material (slurry). Finally, the binder was removed and sintered to give the component its final properties [4].

The long-term prognosis of dental crowns depends on many aspects, with marginal adaptation being one of the most important factors that may lead to clinical failure [5, 6]. A marginal gap is defined as the vertical distance between the finish line of the preparation and the cervical margin of the restoration [7, 8]. This could be measured under high magnification with a stereomicroscope to measure the marginal gap from the crown margin to the finish line [9]. Poor marginal adaptation can lead to plaque accumulation, microleakage, recurrent caries, and periodontal disease [10]. A marginal gap between 50 and 120 µm is considered clinically acceptable [5]. Moreover, the internal fit is an important factor for the success of dental crowns, as deterioration of the internal fit of the crown will lead to a decrease in retention, a lack of rotational stability, and a reduction in fracture toughness [11].

There are also various techniques that can be used to measure the internal fit of dental crowns; such as repeated 3D scanning, which is a non-destructive method but requires special care to avoid errors [12, 13]. The cross-sectional method for cemented restoration is a well-known but destructive method [14, 15]. The triple scan protocol is also a non-destructive technique in which the fitting surface of the crown, the abutment and the crown seated on the abutment are scanned and overlapped using a software that allows measurement of the internal fit [16]. SRT is also a well-known technique for measuring the internal fit and the marginal gap [17–19].

Limited evidence is available on the marginal gap and the internal fit of 3D printed zirconia crowns as it is still a new technology. Therefore, it was the aim of this in vitro study to compare the aforementioned properties of 3D printed zirconia crowns with that of the commonly used CAD/CAM milling technique. The first null hypothesis was that there would be no significant difference in the marginal adaptation between the crowns fabricated by milling or 3D printing techniques. The second null hypothesis postulated that the measurements of internal fit would be similar for both techniques used in this study.

## Methods

A typodont upper premolar tooth was prepared to create a 1 mm rounded deep chamfer with 6° convergence of the axial wall and 1.5 mm occlusal reduction. Addition silicone impression material (elite HD + , Zhermack SPA, Rovigo, Italy) putty soft and light consistency was used to make an impression replica. After the complete setting of the index, an epoxy die (KEMAPOXY 150 3D, CMB, Wadi El Natroun, Egypt) having the same modulus of elasticity as the human dentin was poured and allowed to set until full hardening. Then the epoxy die was scanned with an extra-oral scanner (Medit T500, MEDIT Corp., Seoul, Korea), and the produced STL files were used to digitally design the crowns using CAD software (exocad version 3.0, exocad Gmbh, Darmstadt, Germany) with a 70 µm cement gap starting 1 mm from the finish line margin [20].

A power analysis was designed to obtain sufficient power for a two-sided statistical test of the null hypothesis that there is no difference between the tested groups. Assuming an alpha (α) level of 0.05 (5%), a beta (β) level of 0.2 (20%) (i.e., power = 80%), and an effect size (d = 1.53) calculated based on the results of a previous study [20], the predicted sample size (n) was found to be 16 samples i.e., 8 samples per group. Sample size calculation was performed using G*Power version 3.1.9.4 [21]. Thus, 20 zirconia crowns were fabricated using 3 mol-% yttria-stabilized zirconia using 2 different fabrication techniques with 10 crowns in each group.

In group M, IPS e.max ZirCAD LT (Ivoclar Vivadent, New York, USA) crowns were milled using a milling machine (DGSHAPE DWX-520 milling machine, Roland company, Willich, Germany) followed by sintering in a zirconia furnace (Tabeo, MIHM VOGT, Stutensee Blankenloch, Germany) up to 1530 °C. In group P, Lithoz 210 3Y (Lithoz GmbH, Vienna, Austria) crowns were 3D printed using a CeraFab7500 printer (Lithoz GmbH, Vienna, Austria), several small supports placed perpendicular to the lingual surface to hold and stabilize the crowns on the platform until the printing was complete. Then the printed crowns were heated in 3 consecutive furnaces (Nabertherm oven, Nabertherm GmbH, Lilienthal, Germany) as follows: 1) Preconditioning up to 120 °C for 134 h, 2) transfer to the second oven for debinding at up to 1000 °C for 103 h, and 3) sintering at up to 1450 °C for 17 h.

The internal surface of all crowns was not adjusted after sintering. The crowns were sandblasted using alumina particles of 50 µm at 2 bar for 10 s. The crowns were seated on the same epoxy die and a sharp dental explorer was used to check for proper seating.

### Marginal gap evaluation (vertical marginal gap technique VMGT)

**Fig. 1:**
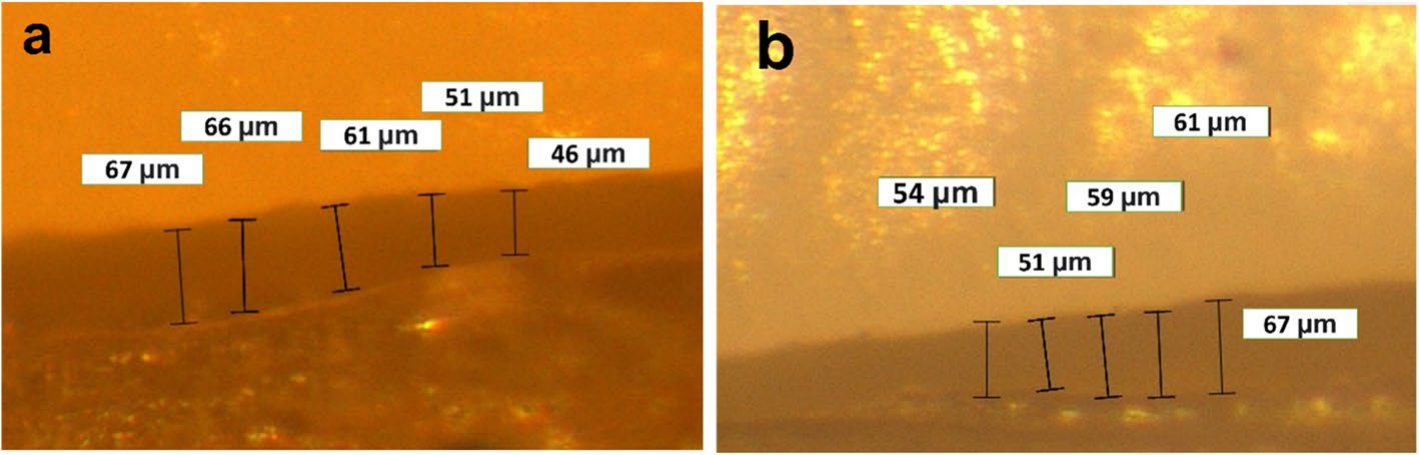
Measurements of marginal gap. **a** Milled zirconia crown. **b** Printed zirconia crown

A stereomicroscope (Wild Lecia M8, Leica Mikrosysteme, Heerbrugg, Switzerland) with a digital camera (Leica DFC 420 C, Leica Mikrosysteme, Wetzlar, Germany) at 50X magnification was used to observe the marginal gap, which was measured using Leica software (Leica LAS AF LITE 4.10.0) as shown in Fig. 1. Calibration protocols of microscope were followed strictly before starting the measurements. The marginal gap was determined using the criteria proposed by Holmes et al. [7] who defined the vertical marginal gap as the distance between the crown margin to the edge of the finish line preparation. For standardization, each axial wall was divided into 3 equal parts, at each part 5 measurements for the marginal gaps were recorded, thus, 15 measurements for each axial wall were obtained, which were averaged to yield a single measurement for each sample [22].

### Internal fit evaluation and marginal gap (Silicone replica technique SRT)

**Fig. 2:**
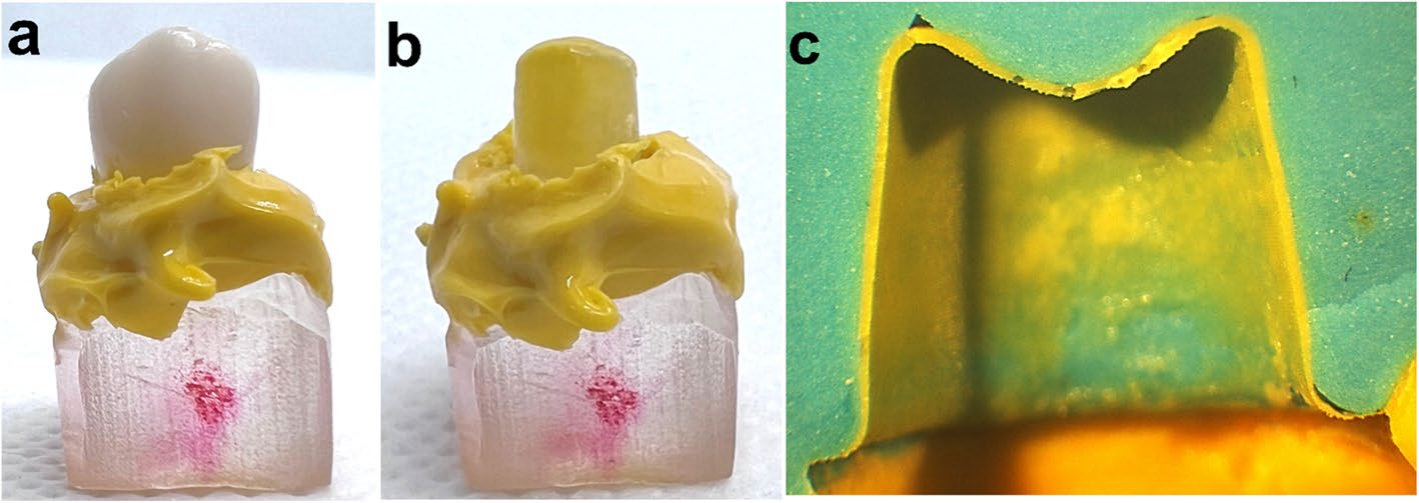
Fabrication of silicone replica. **a** Low viscosity silicone impression material was injected in the fitting surface of the crown and then fitted to the epoxy die. **b** Crown was removed, and the low viscosity impression remains attached to the die. **c** After the putty was added, the replica was removed and cut buccolingually as in the figure, then it was cut mesiodistally

The internal fit was measured using the silicone replica technique in which a low viscosity silicone impression material (Honigum light, DMG, Hamburg, Germany) was injected into the fitting surface of the crown. The crown was then seated over the abutment and pressed for 3 min and 30 s under a 5 kg load until the impression material was fully set according to the manufacturer’s instructions. Subsequently, the crown was removed leaving the light silicone impression on the abutment representing the thickness of the cement space. A putty silicone material (Honigum putty soft, DMG, Hamburg, Germany) was then applied over the remaining light impression on the abutment to overcome the difficulties in handling and cutting the thin thickness of the light body. After setting, the silicone replica was removed and cut into four parts buccopalatally and mesiodistally using surgical blade no. 15 as shown in Fig. 2.

**Fig. 3:**
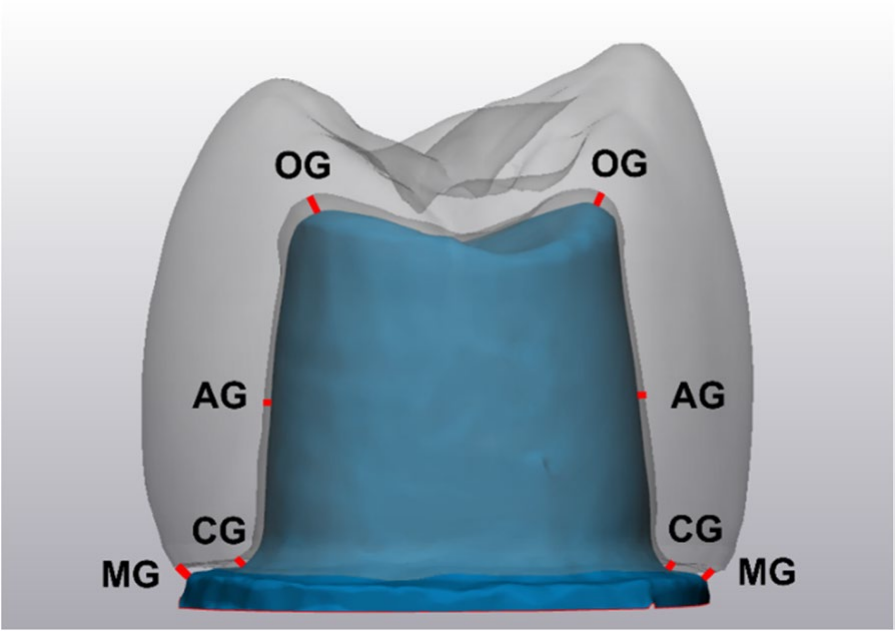
Each section was measured at four different points: Marginal Gap (MG), Cervical Gap (CG), Axial Gap (AG), Occlusal Gap (OG)

**Fig. 4:**
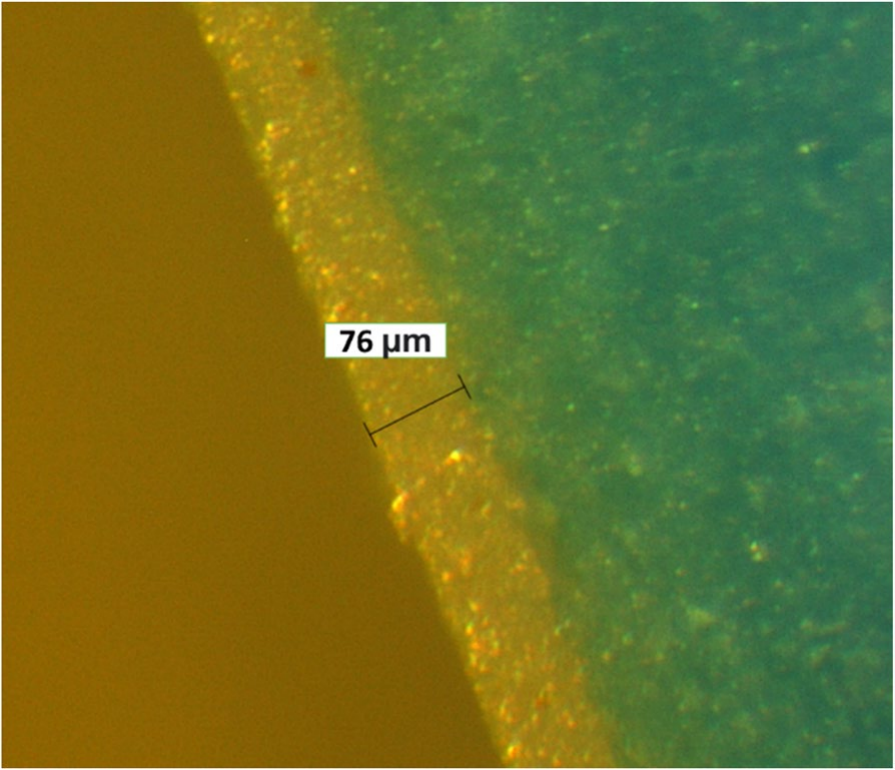
Measuring the AG under 50 X magnification

The thickness of the light body was observed using a stereomicroscope (Wild Lecia M8, Heerbrugg, Switzerland) coupled with a Leica DFC 420 C digital camera (Leica Mikrosysteme, Wetzlar, Germany) at 50X magnification to evaluate the internal fit and the marginal gap. The thickness of the light body silicone was measured at 16 different points, which were divided into 4 groups: Marginal Gap (MG), Cervical Gap (CG), Axial Gap (AG), and Occlusal Gap (OG). The internal fit was evaluated by the CG, AG, and OG [19]. Each point was measured using Leica software (Leica LAS AF LITE 4.10.0) as shown in Figs. 3 and 4.

### Statistical analysis

Numerical data were tested for normality using Shapiro–Wilk's test and represented as mean and standard deviation. Data were then analyzed using independent t-test, reliability analysis was performed with the Intra-class correlation coefficient (ICC). Correlation analysis was performed using Spearman’s rank order correlation coefficient (rs). The significance level was set at *p* < 0.05 for all tests. Statistical analysis was performed with R statistical analysis software (R Core Team 2023) version 4.1.3 for Windows.

## Results

The results of intergroup comparisons for marginal gap are presented in Table 1. It was found that group P had significantly higher mean values of 80 µm compared to group M that showed a mean marginal gap of 60 µm (*p* < 0.05).

**Table 1:** Intergroup comparisons of marginal gap (in µm) of the 3D printed (P) and milled (M) groups

*Point*	*Marginal gap in µm (Mean* ± *SD)*		*t-value*	*p*-*value*
	***Group M***	***Group P***		
***Average***	60 ± 20	80 ± 30	**3.59**	< 0.001*

^*^significant (*p* < 0.05)

**Fig. 5:**
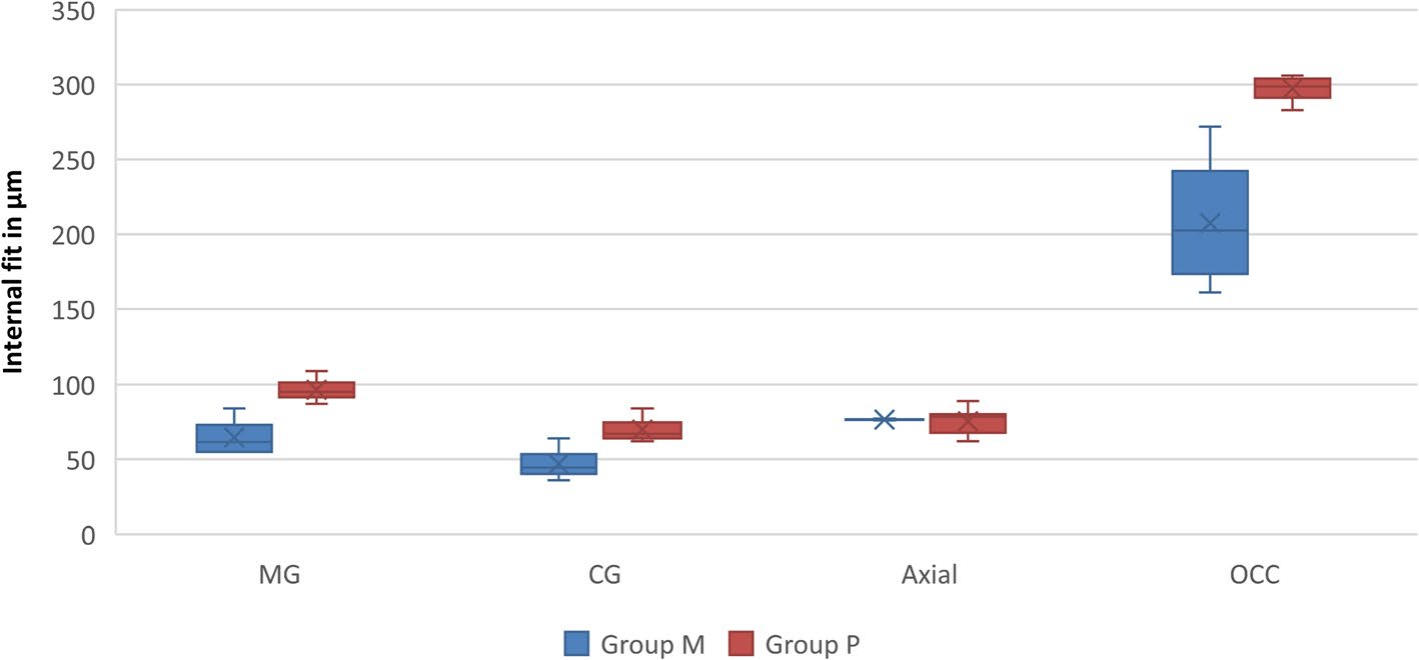
Box plot showing values of internal fit measured at different points

The results of intergroup comparisons of internal fit showed that group P had significantly higher mean values than group M for MG, CG and OCC measurements (*p* < 0.001), while the differences for axial measurements were not statistically significant (*p* > 0.05). The Mean and standard deviations for internal fit are illustrated in Fig. 5.

**Fig. 6:**
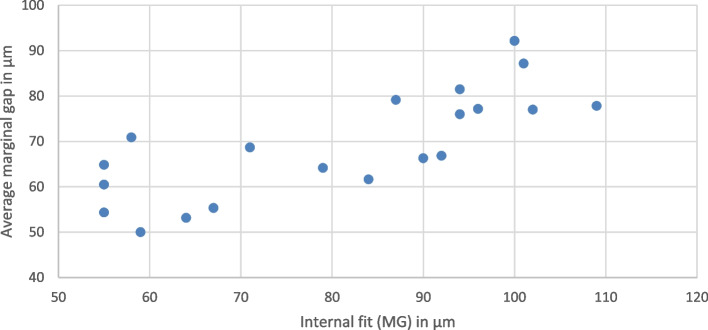
Scatter plot showing the correlation between average marginal gap under stereomicroscope and MG using silicone replica technique

The results of reliability and correlation analyses showed that there was a strong agreement (ICC = 0.715) and positive correlation (rs = 0.774) between the marginal gap (VMGT) using stereomicroscope and the marginal gap using the silicone replica method (MG) (*p* < 0.001) as shown in Fig. 6.

## Discussion

In this study, the first null hypothesis was rejected because the measurements of the marginal gap were lower in the milled group (M) than in the 3D printed group (P). The second null hypothesis was rejected, as the measurements of the marginal gap, cervical gap, and occlusal gap were lower in the milled crowns than in the 3D printed crowns, except for that the axial gap.

A large marginal gap between the restoration and the tooth leads to leakage and recurrent caries. Thus, the presence of a marginal gap is one of the most important aspects to be considered when choosing the method for fabricating a dental crown, especially when new technologies are used. Despite careful preparation, there is always a gap between the margin of a full coverage restoration and the finish line of the prepared tooth. In addition, the internal fit of restoration is important for the retention and resistance of the crown. The literature reports that the normal acceptable marginal gap should be below 120 μm and the occlusal gap between 250 and 300 μm [5, 23].

At the beginning of this research, micro-CT was used to measure the marginal gap and the internal fit, but then it was excluded due to some limitations that made the readings inaccurate. It was extremely difficult to define specific points at the margin of the restoration and the finish line, which could be due to the radiation absorption coefficient and artificial defects caused by the reflection of rays [21, 24]. Accordingly, two of the most common techniques were used to measure the marginal gap and the internal fit. In the first method, a stereomicroscope was used to measure the marginal gap between the crown margin and the finish line [9]. In the second method, the silicone replica technique using light body silicone impression material was used to determine the cement space thickness in order to evaluate the internal fit as well as the marginal gap of the restoration. Many authors compared the use of SRT to other techniques, and they found that it offers reliable results with the advantage of being used in both in vivo and in vitro studies as it is a non-destructive method [21, 25, 26]. The results of this study showed strong agreement between the two methods used in measuring the marginal gap.

The values of the marginal gaps of all zirconia crowns manufactured with both techniques were below 110 μm, thus all the specimens were within the clinically acceptable range. The range of the marginal gap of the milled crowns was 60 ± 20 μm. This is in agreement with the results of many authors who investigated the marginal gap of monolithic zirconia crowns, where the gap ranged from 24 to 110 μm for CAD/CAM restorations [9, 27–30]. Moreover, the marginal gaps of the 3D printed crowns were in the range of 80 ± 30 μm, which is in accordance with Ryu et al. [19] who compared the effect of different build directions on the marginal and internal fits of 3D printed resin crowns.

The mean marginal gap of the milled crowns in this study was significantly lower than that of the 3D printed zirconia crowns. This could be related to over-polymerization of the material during fabrication due to light scattering, which means that more material hardens than intended. However, the manufacturer set a contour offset of 40 µm in the printer software to counteract this effect. Therefore, additional studies are needed to investigate the effect of changing the contour offset value during the manufacturing of zirconia crowns on enhancing the marginal adaptation.

For the SRT, the results of the marginal gap, cervical, and occlusal gap between milled and printed crowns showed that the milled crowns had lower values than the 3D printed crowns. Comparing the marginal gap results of the 3D printed crowns between the two measurement methods used, the values were higher in the SRT. This could be due to the preexisting friction noticed when the 3D printed crowns were seated on the epoxy die during the checking phase so when the light impression was injected into the fitting surface, it caused the increase in the marginal gap, cervical gap and the occlusal gap in 3D printed crowns. This problem can be solved by increasing the cement gap by more than 70 μm by 10–15 μm and testing the results as suggested by Ha et al. [21]. In many studies using the 3D printing technique in the fabrication of resin-based crowns, it was found that the measured internal fits were 3–4 times the cement gap used in designing. Thus, increasing the cement gap value during designing the restoration with the CAD software can improve the fit of the fabricated 3D printed crowns [19, 31].

This study has several limitations. First, the marginal gap and the internal fit of the 3D printed crowns were not measured between the different manufacturing steps. Future studies are recommended to allow fine-tuning of the manufacturing process. Second, the use of natural human teeth instead of resin abutment would simulate the clinical conditions, although resin abutment was used in this study to allow standardization.

Additional studies are needed to determine the optimal cement space value for the CAD design, which is required to reduce marginal gap without compromising the internal fit and to provide maximum retention and resistance for better clinical outcomes.

## Conclusions

The 3D printed zirconia crowns showed higher values for marginal gap and internal fit compared to the milled zirconia crowns, but within the clinically acceptable range. The 3D printing technique showed promising results within the clinically acceptable range for marginal gap and internal fit. Thus, 3D printed monolithic zirconia crowns can be considered a clinically acceptable alternative in terms of marginal adaptation and the internal fit. The evaluation of marginal gap could be measured with both the VMGT and the SRT, with both techniques showing reliable and comparable results.

## Data Availability

The datasets supporting the conclusions of this article are included within the article.

## Abbreviations

VMGT: Vertical marginal gap technique
SRT: Silicone replica technique
CAD/CAM: Computer aided designing / Computer aid manufacturing
AM: Additive manufacturing
LCM: Lithography-based Ceramics Manufacturing technique
DLP: Digital light processing
MG: Marginal gap
CG: Cervical gap
AG: Axial gap
OCC: Occlusal gap
